# Cardiovascular Responses and Energy Expenditure During a Standardized Training Programme in Three-Year-Old Czech Warmblood Stallions

**DOI:** 10.3390/ani16203292

**Published:** 2026-10-19

**Authors:** Daniela Kacetlová, Hana Dočkalová, Tomáš Kopec, Daria Baholet Bátik, Andrej Bátik

**Affiliations:** 1 Department of Animal Breeding, Faculty of AgriSciences, Mendel University in Brno, 61300 Brno, Czech Republic; xbajkova@mendelu.cz (D.K.); tomas.kopec@mendelu.cz (T.K.); 2 Department of Animal Nutrition and Forage Production, Faculty of AgriSciences, Mendel University in Brno, 61300 Brno, Czech Republic; 3 Department of Animal Morphology, Physiology and Genetics, Faculty of AgriSciences, Mendel University in Brno, 61300 Brno, Czech Republic; andrej.batik@mendelu.cz

**Keywords:** Czech Warmblood, energy expenditure, exercise workload, heart rate, young stallions

## Abstract

Background: Early training represents an important period in the development of young sport horses, yet field-based information on cardiovascular responses and estimated energy expenditure across different exercise modalities remains limited. Objectives: This study aimed to characterize heart rate (HR) responses and estimate energy expenditure in three-year-old Czech Warmblood stallions during an 18-week standardized training programme, with emphasis on exercise modality and temporal variation. Hypothesis: We hypothesized that HR responses and estimated energy expenditure would differ among exercise modalities and vary throughout the training period. Methods: Fourteen three-year-old Czech Warmblood stallions were monitored over 18 weeks, comprising 1003 training sessions across six exercise modalities. Session mean, peak, and minimum HR were recorded, and energy expenditure was estimated using HR-based equations and National Research Council (NRC) recommendations. Linear mixed-effects models assessed associations with exercise modality and monitoring week, accounting for repeated observations within horses. Results: Exercise modality was associated with mean HR (χ^2^ = 284.64, *p* < 0.001) and peak HR (χ^2^ = 122.08, *p* < 0.001), but not minimum HR (*p* = 0.066). Monitoring week was associated with all three HR outcomes (*p* < 0.001), with significant modality × week interactions. Estimated energy expenditure per session differed among exercise modalities (*p* < 0.001), with the highest model-based values observed during free jumping and horse walker exercise and the lowest during lunging. NRC-based estimates of daily energy expenditure (113.87 ± 11.73 MJ/day) exceeded estimates obtained using the Coenen HR (100.33 ± 6.23 MJ/day) and Coenen O_2_ (95.78 ± 6.68 MJ/day) methods. Conclusions: Cardiovascular responses and estimated energy expenditure differed among exercise modalities, while temporal HR variation could not be attributed solely to cardiovascular adaptation. Assessment of training load should consider both exercise characteristics and session duration. HR monitoring provides complementary information under field conditions, but HR-derived energy estimates should not be regarded as independently validated measures of energy requirements.

## Simple Summary

Standardized training and performance testing are important in the selection of young sport horses, but their physiological demands under field conditions remain insufficiently described. We monitored heart rate and estimated energy expenditure in 14 three-year-old Czech Warmblood stallions during an 18-week training programme. Heart rate responses differed among exercise modalities and varied over time, although these changes could not be attributed solely to training adaptation. Prolonged lower-intensity activities contributed substantially to estimated energy expenditure, while daily energy estimates differed between calculation methods. These findings may support a more comprehensive evaluation of training demands alongside performance-based assessments.

## 1. Introduction

Training and physical exercise induce coordinated cardiovascular responses aimed at maintaining homeostasis under increased metabolic demand. In horses, exercise and transport represent important physiological stressors that elicit endocrine and inflammatory responses, indicating that workload affects multiple physiological systems beyond locomotion alone [1,2]. During early conditioning, young horses are exposed to novel workloads and environmental stimuli that result in pronounced changes in heart rate (HR) dynamics, making this period particularly relevant for the assessment of cardiovascular adaptation [3]. However, cardiovascular responses during initial training may also reflect psychological stress associated with unfamiliar procedures and human–horse interactions, rather than physical workload alone [4].

In the Czech Republic, the evaluation of young Warmblood horses includes performance testing, with three-year-old stallions also assessed following a period of rearing and training at specialized testing facilities [5]. These systems have demonstrated moderate heritability of performance and conformation traits and support genetic improvement and management decisions [6,7,8]. In addition, sex-related differences in competition outcomes have been reported in young sport horses, suggesting that physiological responses to training may differ even at early ages [9,10].

Heart rate monitoring is a widely used, non-invasive method for quantifying cardiovascular load and training responses in equine athletes. Continuous HR measurement enables objective assessment of exercise intensity and allows estimation of energy expenditure under field conditions [11,12]. Recent advances in wearable technologies have improved the feasibility of cardiovascular monitoring during exercise, supporting objective assessment of physiological responses in sport horses [13].

A major challenge in interpreting HR responses under field conditions is substantial inter- and intra-individual variability, which may obscure true training effects. Longitudinal studies in young sport horses have demonstrated that HR patterns are strongly influenced by workload composition and exercise modality and that cardiovascular responses may differ among horses exposed to similar training regimens [14,15]. Therefore, standardized exercise protocols are essential for distinguishing workload-specific physiological responses from variability caused by inconsistent workload exposure [12,16].

Despite increasing use of HR monitoring in equine training, most longitudinal field studies have focused on racehorses or isolated exercise tests, such as incremental field or treadmill protocols [17,18,19]. In contrast, fewer studies have quantified HR dynamics and HR-derived energy expenditure across the range of exercise modalities used in routine training typically applied during the early preparation of Warmblood stallions for performance selection [15,20]. Consequently, the contribution of individual workload types to variability in physiological responses within a standardized training framework remains insufficiently characterized.

In addition to their role in training, standardized station performance tests represent a key component of selection systems for young sport horses. However, evaluation within these systems is largely based on performance outcomes and subjective assessment, while objective physiological indicators of workload and adaptation are rarely incorporated. Continuous HR monitoring during standardized training therefore provides an opportunity to complement traditional evaluation by delivering objective data on cardiovascular load and energy expenditure under real training conditions. Although such measurements may not be feasible in routine practice for all horses, they can provide valuable insight into workload characteristics and physiological demands, improving interpretation of performance test results and supporting more informed decisions in training management, nutritional planning, and equine welfare assessment.

Therefore, the present study aimed to evaluate HR responses and estimate energy expenditure in three-year-old Czech Warmblood stallions during an 18-week standardized training programme, with particular emphasis on the effects of exercise modality and temporal changes throughout the monitoring period.

## 2. Materials and Methods

### 2.1. Ethical Approval

All procedures involving animals were conducted in accordance with Czech national legislation (Act No. 246/1992 Coll.) and EU Directive 2010/63/EU. The study involved only non-invasive heart rate monitoring performed during routine standardized training and did not alter or interfere with the animals’ standard management, handling, or training procedures. Therefore, no separate study-specific ethical approval was issued. The research project was assessed and approved within the applicable institutional framework of Mendel University in Brno, and the work involving animals was conducted under the relevant general authorization (No. 57890/2012-MZE-17214).

### 2.2. Animals, Housing, and Feeding Regimen

Fourteen clinically healthy three-year-old Czech Warmblood stallions (*n* = 14) were included in the study. Mean body weight was 541 ± 38.5 kg and body condition score ranged from 5.5 to 6 (Table 1), which is considered optimal moderate condition [21]. Horses were housed in individual boxes with free access to water and salt lick (halite without additives) and were fed a standardized ration formulated according to NRC recommendations; the composition and estimated energy value of the ration are presented in Table 2.

### 2.3. Training Activities

The monitored training activities were:

1. Horse walker: walking or trotting in a mechanical walker.
2. Lunging: circular exercise in walk, trot, and canter.
3. Lunging with a saddle: progressive habituation to load during lunging.
4. Work under saddle: ridden exercise in all gaits.
5. Free jumping: repeated jumping sequences including warm-up and cool-down on the walker.
6. Test: free-jumping session with blood sampling before and after exercise.

Following arrival at the stud farm, the stallions underwent an initial period of habituation to the new environment before HR data collection began. During the first two weeks of the 18-week monitoring period, the horses were introduced to regular work through lunging and horse walker exercise while also being habituated to the heart rate monitoring equipment. Training followed a general plan, with the choice and frequency of exercise modalities adjusted to the stage of breaking-in and the horses’ progress. Lunging with a saddle represented a transitional stage during habituation to saddling and preparation for ridden work. As training progressed, ridden sessions became more frequent. Consequently, the substantially different numbers of observations for lunging with a saddle and ridden work reflected their different roles and periods of use within the training process.

For organizational purposes, the horses were allocated to three groups of five, five, and four animals according to arena capacity and rider availability. These groups did not represent different experimental training regimens. Horses within a group generally performed the same exercise modality on a given day, although individual differences occurred on some days. Training was generally conducted on weekdays, with weekends usually reserved for rest. The indicative weekly schedule is presented in Table 3. However, the allocation of activities to particular days varied during the training period.

The duration of individual exercise modalities differed by design and reflected routine training practice (mean ± SE: 22.67 ± 0.98 to 97.96 ± 2.11 min, depending on activity type). These differences in duration were inherent to the exercise modalities as performed in routine training and should be considered when interpreting comparisons of HR responses and estimated energy expenditure. The duration of the warm-up and cool-down phases was not fixed but varied according to the type and duration of each training activity. For the purposes of this study, one unit of workload was defined as one complete training session, including warm-up and cool-down.

### 2.4. Heart Rate Monitoring

Heart rate was continuously recorded during all training activities from the start of warm-up to the end of cool-down using a heart-rate monitoring system [22]. The system consisted of an HR sensor, an equine electrode belt, and a GPS watch, with data transferred to a computer and processed using the manufacturer’s software. The electrode belt was moistened and contact areas dampened to ensure conductivity. During all activities, including free jumping, data transmission was wireless; the watch was attached to the bridle during exercise.

Only sessions with complete HR recordings covering the entire training session, including warm-up and cool-down, were included in the analysis. Recordings with signal loss or gaps were excluded. Complete recordings were not additionally screened for transient HR spikes or other potential artefacts, and no minimum duration criterion was applied to recorded peak values. For each included session, minimum HR, mean HR, and session peak HR were extracted. Session peak HR was defined as the highest recorded HR value within that session and was not considered a measurement of physiological maximal heart rate (HRmax).

### 2.5. Estimation of Digestible Energy

The digestible energy (DE) in the feed was determined according to the formula [23]: DE (MJ/kg dry matter) = −3.6 + 0.211 × (% crude protein) + 0.421 × (% ether extract) + 0.015 × (% crude fibre) + 0.189 × (% NFE).

### 2.6. Estimation of Energy Output

Energy output was calculated according to [24]: ME (J) = 0.0566 × HR^1.9955^ × time × body weight. Metabolizable energy was converted to DE using the equation of Ebert and Moore-Colyer [11].

Oxygen consumption was estimated according to Coenen [25]: Estimated oxygen consumption (mL O_2_/kg BW/min) = 0.0019 × HR^2.0653^. The resulting values were adjusted for total body mass (including rider and equipment), converted to litres, and expressed as energy expenditure using a caloric equivalent of 4.86 kcal per litre of oxygen [26]. Estimated energy expenditure was subsequently converted to megajoules and multiplied by total training time.

Oxygen consumption was estimated from HR and was not measured directly by respiratory gas analysis. Both energy expenditure estimates were therefore derived from the same HR recordings and should be regarded as alternative calculation approaches rather than independent physiological measurements.

Daily energy requirements were calculated according to NRC [26] guidelines. Horses were classified as performing light (mean HR < 90 bpm) or moderate work (mean HR ≥ 90 bpm), with energy requirements increased by 20% or 40% above maintenance, respectively. This classification was used to individualize NRC-based estimates according to physiological response.

### 2.7. Data Evaluation and Statistics

Heart rate outcomes (session mean HR, session peak HR, and session minimum HR) and estimated energy expenditure per training session (Coenen HR and Coenen O_2_ methods) were analysed separately using linear mixed-effects models to account for repeated observations within individual horses. Energy expenditure was log-transformed before analysis because of its right-skewed distribution and the residual characteristics of models fitted on the original scale. Exercise modality and monitoring week were included as fixed effects, while horse identity was included as a random intercept. In the primary models, week was treated as a categorical factor to evaluate temporal variation without assuming a linear pattern across the 18-week monitoring period. Fixed effects were evaluated using approximate Wald chi-square tests. Additional models treated week as a continuous variable to estimate the linear temporal trend after adjustment for exercise modality; slopes are reported with 95% confidence intervals. For energy expenditure, linear temporal changes were expressed as percentage changes per week. Back-transformed estimated marginal means (geometric means) and 95% confidence intervals were calculated by averaging equally across monitoring weeks on the log scale. Supplementary energy models additionally included log-transformed session duration as a covariate.

The exercise modality × week interaction was examined in a supplementary mixed-effects analysis of the HR outcomes, restricted to modality–week combinations represented in the dataset. These models included a random intercept for horse and allowed residual variances to differ among exercise modalities. Models with and without the interaction were compared using maximum likelihood and approximate likelihood-ratio tests. A sensitivity analysis additionally included a random intercept for horse–week combinations. Mixed-effects analyses were implemented in Python (version 3.13.5) with NumPy (version 2.3.5), SciPy (version 1.17.0), and statsmodels, as appropriate. Statistical significance was set at *p* < 0.05.

The original analyses of session duration and distance covered were performed using STATISTICA.CZ 12.0, with additive analysis of variance followed by Scheffé’s post hoc test. These analyses were retained as exploratory comparisons and did not account for the repeated-measures structure of the dataset. Their significance tests should therefore be interpreted cautiously. Original additive-model summaries of energy expenditure by individual horse are retained in the Supplementary Materials for descriptive purposes; inferential conclusions for energy expenditure are based on the mixed-effects models.

## 3. Results

### 3.1. Heart Rate Responses Across Exercise Modalities and Monitoring Weeks

The analysis included 1003 training sessions from 14 horses monitored over 18 weeks. In the primary linear mixed-effects models, exercise modality was associated with session mean HR and session peak HR (both *p* < 0.001), whereas the overall modality effect on session minimum HR was not statistically significant (*p* = 0.066). Monitoring week, treated as a categorical factor, was associated with all three HR outcomes (all *p* < 0.001; Table 4).

When week was treated as a continuous variable, session mean HR increased by an estimated 0.321 bpm per week (95% CI: 0.111 to 0.531; *p* = 0.0027), session peak HR decreased by 0.423 bpm per week (95% CI: −0.764 to −0.082; *p* = 0.0152), and session minimum HR increased by 0.672 bpm per week (95% CI: 0.485 to 0.860; *p* < 0.001), after adjustment for exercise modality.

The supplementary mixed-effects analysis identified an exercise modality × week interaction for session mean HR (*p* = 0.0023), session peak HR (*p* = 0.0030), and session minimum HR (*p* = 0.0054). Thus, differences among exercise modalities varied across monitoring weeks. The interaction remained statistically significant for all three outcomes in the sensitivity analysis, including an additional random intercept for horse–week combinations. The highest recorded HR was 229 bpm. Descriptive summaries indicated higher mean HR during lunging and lunging with a saddle and lower mean HR during horse walker exercise (Supplementary Table S2). Session duration and distance covered also varied among exercise modalities, while weekly summaries documented temporal variation in these measures and HR (Supplementary Table S3). Individual horse-level HR summaries are provided in Supplementary Table S1.

### 3.2. Comparison of Energy Expenditure Estimation Methods

**Figure 1:**
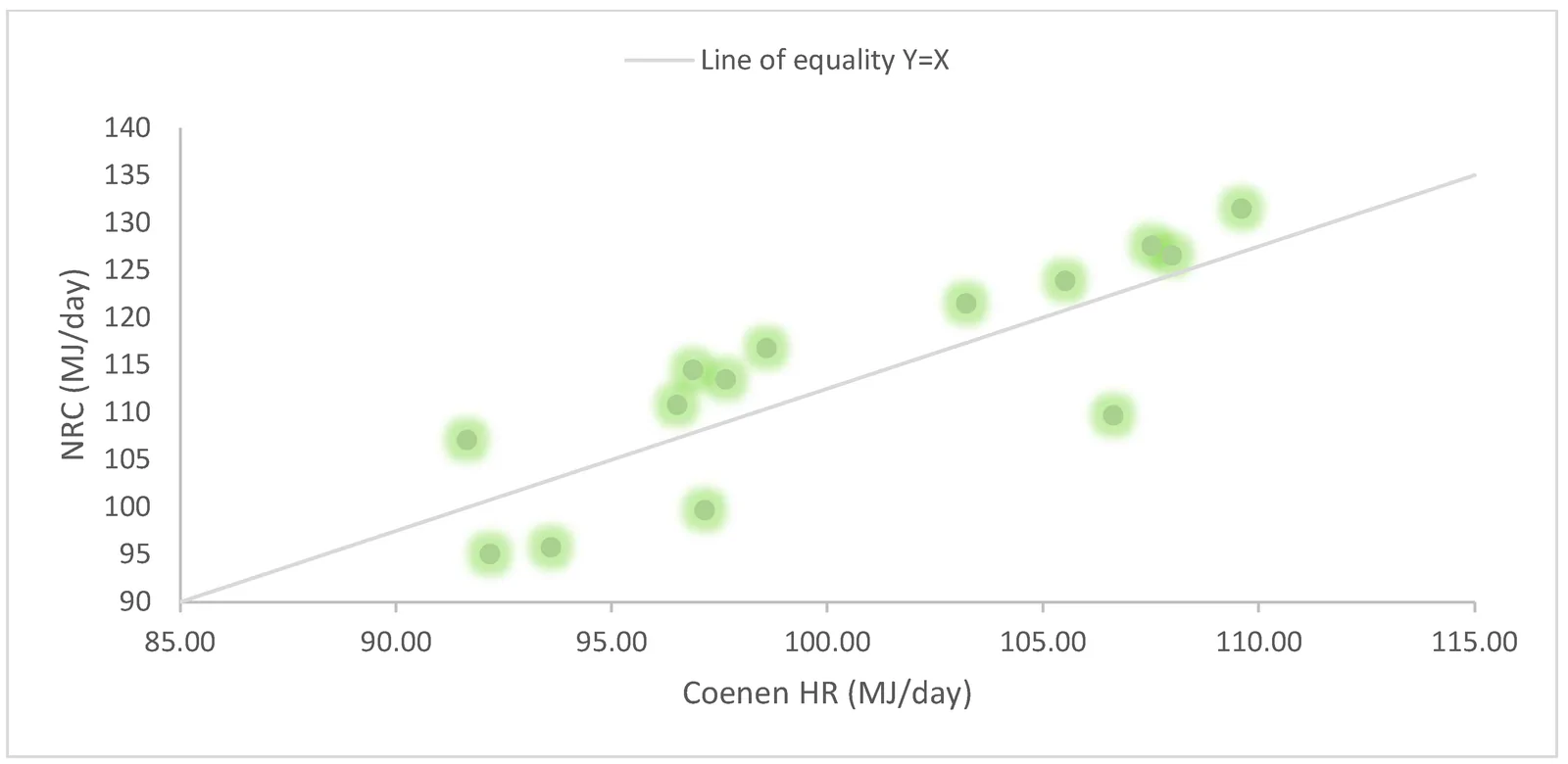
Relationship between daily energy expenditure calculated using the Coenen heart rate method and NRC-based estimates in 14 three-year-old Czech Warmblood stallions. Each point represents the mean daily value for one horse. The grey line indicates the line of equality (y = x). NRC values were consistently higher than Coenen-derived estimates across all individuals.

Estimated daily energy expenditure differed among calculation methods (Table 5). NRC-based estimates (113.87 ± 11.73 MJ/day) were higher than estimates obtained using the Coenen HR (100.33 ± 6.23 MJ/day) and Coenen O_2_ methods (95.78 ± 6.68 MJ/day). Both HR-derived methods showed lower inter-individual variability, as reflected by their smaller SD values and narrower ranges. The relationship between NRC-based and Coenen HR estimates is shown in Figure 1. Individual horse-level summaries of estimated energy expenditure per training session are provided in Supplementary Table S4.

### 3.3. Energy Expenditure Across Workload Types

Estimated energy expenditure per training session differed among exercise modalities in the linear mixed-effects models for both the Coenen HR method (χ^2^ = 216.09, df = 5, *p* < 0.001) and the Coenen O_2_ method (χ^2^ = 199.39, df = 5, *p* < 0.001). Monitoring week was also associated with estimated energy expenditure when treated as a categorical factor (Coenen HR: χ^2^ = 29.38, df = 17, *p* = 0.0312; Coenen O_2_: χ^2^ = 29.28, df = 17, *p* = 0.0320).

The highest model-based geometric mean estimates were observed during free jumping and horse walker exercise, whereas lunging showed the lowest estimated energy expenditure (Table 6). These estimates describe energy expenditure per recorded training session and therefore reflect differences in both cardiovascular response and session duration.

After additional adjustment for log-transformed session duration, the association between exercise modality and estimated energy expenditure remained statistically significant for both methods (Coenen HR: χ^2^ = 131.78, df = 5, *p* < 0.001; Coenen O_2_: χ^2^ = 131.79, df = 5, *p* < 0.001). No significant linear temporal trend was detected when monitoring week was treated as a continuous variable. The estimated weekly changes were −0.31% (95% CI: −0.94% to 0.32%; *p* = 0.336) for Coenen HR and −0.29% (95% CI: −0.93% to 0.36%; *p* = 0.381) for Coenen O_2_.

## 4. Discussion

The present study characterized cardiovascular responses and estimated energy expenditure during an 18-week training programme in three-year-old Czech Warmblood stallions under field conditions. Heart rate responses and estimated energy expenditure differed among exercise modalities, while temporal variation in HR was also observed throughout the monitoring period. However, the relative contributions of training progression, habituation, and cardiovascular adaptation could not be determined. NRC-based estimates of daily energy expenditure were consistently higher than HR-derived estimates, reflecting differences in the underlying calculation approaches rather than demonstrating the accuracy of either method.

### 4.1. Heart Rate Responses During Early Training

The mean HR values observed in the study were within the range reported for young sport horses during early training, confirming that moderate cardiovascular load is typical for this stage of conditioning [14,15,19]. Previous studies have reported training-related changes in HR parameters during early conditioning [14,15]. Temporal changes were also detected in the present study, although their interpretation must account for the changing exercise demands during progressive training.

The mixed-effects analyses identified temporal changes in HR after adjustment for exercise modality. The estimated linear trends differed among outcomes, with increases in session mean and minimum HR and a decrease in session peak HR. However, the exercise modality × week interactions indicate that a single overall slope does not fully describe the temporal pattern across activities. These findings cannot establish whether cardiovascular fitness improved or declined, because the horses were undergoing progressive training rather than repeated assessments at a constant workload. Changes in exercise demands and habituation may therefore have contributed to the observed temporal variation alongside cardiovascular adaptation.

Individual variation in HR responses may be influenced by fitness, motor coordination, temperament, and habituation to training. Studies in young sport horses have demonstrated that cardiovascular responses during initial training reflect both physical demands and psychological stress, as indicated by changes in HR, heart rate variability (HRV), and cortisol [3,4]. In Warmblood show jumping horses, the combined assessment of HR and blood lactate has provided additional information on exercise intensity and post-exercise recovery [20].

Peak HR values exceeding 210 bpm were recorded across exercise modalities. Although brief elevations in HR may occur during demanding phases of exercise [18,19], isolated peak values in the present study cannot be interpreted unequivocally as evidence of high-intensity effort because transient recording artefacts were not systematically assessed. Session peak HR should therefore be considered alongside mean HR and the characteristics of each exercise modality.

### 4.2. Effects of Workload Type on Physiological Responses

The highest mean HR was observed during lunging with a saddle (102.6 ± 2.9 bpm), whereas horse walker exercise showed the lowest values (71.2 ± 2.2 bpm). Similar modality-specific differences have been reported in young sport horses, where higher HR values were observed during more demanding exercise types compared with lower-intensity work [15,19]. For example, Siegers et al. [15] reported higher HR during circular and backing work compared with straight-line exercise in Friesian stallions, while Massie et al. [19] observed elevated HR during sport-specific tasks compared with routine ridden work. Recent studies have further demonstrated the use of heart rate responses for assessing physiological workload across different training activities and exercise modalities [3,27].

The energy estimates illustrate why session mean HR should be considered together with exercise duration when describing training demands. Horse walker exercise was associated with relatively low mean HR but comparatively high estimated energy expenditure per session, whereas free jumping also showed high estimated energy expenditure. These observations are consistent with the different combinations of session duration and cardiovascular response across exercise modalities. Because estimated energy expenditure depends on both HR and session duration, these findings do not constitute independent physiological validation. Nevertheless, the association with exercise modality remained significant after adjustment for session duration.

### 4.3. Energy Expenditure and Estimation Methods

Consistent differences were observed between methods used to estimate energy expenditure, with NRC-based values exceeding those obtained using HR-derived methods. This discrepancy highlights differences between the estimation approaches but cannot establish the accuracy of either approach without an independent measurement of energy expenditure. Similar differences between guideline-based and field-derived estimates have been reported in performance horses, where physiological measurements indicated deviations from standardized recommendations [11].

Energy expenditure and metabolic demands vary across different types of equine exercise [28], while substantial variation in metabolic responses depending on exercise type and intensity has also been observed [29].

In addition, experimental studies have shown that both exercise characteristics and training conditions can influence metabolic responses and performance outcomes in horses [30,31]. Recent findings in young sport horses further demonstrate that structured training can influence energy expenditure and locomotor efficiency [32].

Differences between estimation methods likely reflect the assumptions underlying each approach. NRC recommendations are based on standardized workload categories and were developed largely from studies involving light horse breeds [26]. Consequently, breed-related differences may contribute to discrepancies when these recommendations are applied to young Warmblood horses, although such effects were not directly evaluated in the present study. In contrast, both Coenen estimation methods use HR-derived inputs and therefore share important methodological assumptions. Previous studies have shown that HR–VO_2_ relationships can be used to estimate energy expenditure at the individual level [33]. In addition, experimental work has demonstrated that the duration and structure of exercise contribute substantially to total energetic demands in horses [34].

This study has several limitations. The relatively small number of horses (n = 14) limited statistical power and the precision of estimates, particularly for subtle temporal effects. Although mixed-effects models accounted for repeated observations within horses, statistical inference should be interpreted cautiously given the limited number of animals. The first two weeks of monitoring included habituation to the HR monitoring equipment and introduction to regular work; therefore, early HR changes could not be attributed exclusively to exercise or training adaptation. Although recordings with signal loss or gaps were excluded, complete recordings were not screened for transient artefacts, and isolated extreme HR values, particularly during low-intensity activities, should be interpreted cautiously. Energy expenditure was estimated rather than directly measured, and HR-based calculations may not adequately capture brief anaerobic efforts. Both Coenen methods relied on the same HR recordings, with oxygen consumption estimated mathematically; therefore, agreement between them does not constitute independent validation or establish their accuracy relative to NRC-based recommendations. The study included only three-year-old intact stallions, and potential influences of emotional arousal, stress, environmental stimuli, and interactions with handlers or riders on HR were not systematically assessed. Previous research has demonstrated that cardiovascular responses during the initial training of young sport horses may also reflect psychological stress rather than physical workload alone [4]. Exercise modalities also differed in their mechanical characteristics, human interaction, and session duration, which could not be fully disentangled from the physiological responses observed under practical training conditions. Consequently, differences among modalities should not be attributed solely to exercise intensity, and the findings should not be generalized directly to horses of other ages, sexes, or breeds.

Assessment of training load in young sport horses should consider exercise modality, duration, and cardiovascular response rather than relying on a single indicator. HR monitoring can provide complementary information on training demands under field conditions, but its interpretation requires consideration of behavioural influences and the assumptions underlying energy expenditure estimates. The present findings support a more differentiated assessment of routine training activities without establishing the superiority of HR-derived estimates for nutritional planning.

## 5. Conclusions

This study demonstrates that heart rate responses and estimated energy expenditure during early training in three-year-old Czech Warmblood stallions vary among exercise modalities and reflect differences in the characteristics and duration of individual training sessions. Temporal changes in HR were also observed, although these cannot be attributed solely to cardiovascular adaptation. Heart rate responses did not consistently reflect total estimated energy expenditure per session, highlighting the limitations of using HR as a sole indicator of training load. Prolonged low-intensity activities contributed substantially to estimated energy expenditure because of their duration. NRC-based estimates of daily energy expenditure were higher than HR-derived estimates, although these differences do not establish the greater accuracy of either approach. For trainers and riders, these findings emphasize the importance of considering exercise type, cardiovascular response, and session duration when planning and evaluating training workloads in young horses. For veterinarians, HR monitoring may provide complementary information on cardiovascular responses but should not be interpreted as a standalone measure of fitness or welfare.

## Figures and Tables

**Table 1:** Characteristics of the experimental horses.

Characteristics	Value
Age (years)	3
Weight (kg)	541 ± 38.5
Body Condition Score (1–9)	5.5–6.0

**Table 2:** Nutrient composition of feed components.

	Rolled Oat	Extruded Barley	Granulated Feed Mixture	Grass Hay	Grass Haylage	Total Daily Ration
Feed ration (kg/day)	1.0	1.5	2.0	10.0	6.0	20.5
Dry matter (g)	918.9	916.4	932.8	931.3	705.5	17,705.1
Crude fibre (g)	121.1	76.0	91.3	341.0	186.4	4946.1
Crude fat (g)	30.5	34.5	50.1	11.8	21.8	431.3
Crude protein (g)	122.9	136.6	172.1	99.6	89.2	2203.2
Crude ash (g)	20.3	37.4	94.6	70.4	84.9	1479.0
NFE (g)	624.1	631.9	524.7	408.5	323.2	8645.6
Adl-lignin (g)	30.0	29.4	22.9	42.1	32.4	735.3
DE (MJ)	11.0	11.4	11.1	6.5	2.9	132.7

Nutrient contents are expressed per kilogram of feed as offered. Total daily intakes were calculated by multiplying the nutrient content of each feed component by its daily inclusion rate and summing across all components.

**Table 3:** Indicative weekly training schedule.

Day	Exercise Modality	Approximate Planned Duration
Monday	Free jumping	60 min
Tuesday	Ridden work or lunging	40 min
Wednesday	Ridden work	40 min
Thursday	Lunging or horse walker exercise	40 min
Friday	Ridden work	40 min
Weekend	Rest	—

Activity allocation varied with training progression. Durations represent approximate planned values rather than measured session means.

**Table 4:** Results of the primary linear mixed-effects models and supplementary interaction analysis for heart rate outcomes.

Model Term or Estimate	Session Mean HR	Session Peak HR	Session Minimum HR
Exercise modality: χ^2^ (df = 5)	284.64	122.08	10.35
*p*-value	<0.001	<0.001	0.066
Monitoring week, categorical: χ^2^ (df = 17)	104.28	44.70	118.99
*p*-value	<0.001	<0.001	<0.001
Linear weekly change, bpm/week (95% CI)	0.321 (0.111–0.531)	−0.423 (−0.764–−0.082)	0.672 (0.485–0.860)
*p*-value for linear weekly change	0.0027	0.0152	<0.001
Exercise modality × week: likelihood-ratio χ^2^ (df = 40)	70.00	68.98	66.45
*p*-value for interaction	0.0023	0.0030	0.0054

Primary models included exercise modality and monitoring week as fixed effects and horse as a random intercept. Categorical week effects and linear weekly changes were evaluated in separate models. Linear slopes describe temporal associations after adjustment for exercise modality and should not be interpreted as direct estimates of cardiovascular adaptation. Interaction results originate from the supplementary mixed-effects analysis allowing residual variances to differ among exercise modalities. CI, confidence interval; df, degrees of freedom.

**Table 5:** Daily energy expenditure calculated by three different methods in 14 three-year-old Czech Warmblood stallions.

Method	Mean (MJ/day)	SD	Min	Max
Coenen HR	100.33	6.23	91.64	109.60
Coenen O_2_	95.78	6.68	86.48	106.08
NRC	113.87	11.73	95.10	131.50

Values were calculated from the mean daily estimated energy expenditure for each of the 14 horses. SD represents the standard deviation among horses and describes inter-individual variability.

**Table 6:** Model-based geometric mean estimated energy expenditure per training session (MJ/load) according to exercise modality.

Workload	n	Coenen HR (95% CI)	Coenen O_2_ (95% CI)
Walker	58	14.45 (12.42–16.81)	12.39 (10.64–14.44)
Lunging	261	7.71 (6.98–8.50)	6.78 (6.15–7.48)
Lunging + saddle	41	12.28 (10.12–14.90)	10.84 (8.91–13.18)
Saddle	424	11.88 (10.84–13.01)	10.39 (9.49–11.37)
Test	59	11.78 (10.09–13.75)	10.22 (8.75–11.94)
Free jumping	160	14.50 (12.99–16.19)	12.58 (11.26–14.04)

Values are back-transformed estimated marginal means (geometric means) with 95% confidence intervals from log-linear mixed-effects models including exercise modality and monitoring week as fixed effects and horse identity as a random intercept. Estimates were averaged equally across the 18 monitoring weeks. n indicates the number of recorded training sessions. Confidence intervals represent uncertainty in model-based estimates and should not be interpreted as the standard deviation of observations or animals.

## Data Availability

The data presented in this study are openly available in [ZENODO] at [https://zenodo.org/records/21479575, accessed on 1 September 2026], reference number [21479575].

## Supplementary Materials

The following supporting information can be downloaded at https://www.mdpi.com/article/10.3390/ani16203292/s1, Table S1: Descriptive statistics of training sessions by individual horse; Table S2: Descriptive statistics of training sessions by exercise modality; Table S3: Descriptive statistics of training sessions by monitoring week; Table S4: Estimated energy expenditure per unit of workload (MJ/load) calculated by the Coenen HR and Coenen O_2_ methods in 14 three-year-old Czech Warmblood stallions.
