# Supplementary material for: Cardiovascular Responses and Energy Expenditure During a Standardized Training Programme in Three-Year-Old Czech Warmblood Stallions
Source: Animals (Basel). 2026 Oct 19;16(20):3292. doi: 10.3390/ani16203292 (PMC13700651; doi:10.3390/ani16203292)
Supplement: Supplementary file 1 [file animals-16-03292-s001.zip › animals-4571553-supplementary.pdf]

Supplementary Table S1. Descriptive statistics of training sessions by individual horse.

Horse	*n*	*Time (min)*	*Distance (km)*	*HR average (bpm)*	*Session peak HR (bpm)*	*Highest recorded HR (bpm)*	*HR min. (bpm)*
1	72	51.40 ± 1.87	2.81 ± 0.13	89.48 ± 1.90	157.89 ± 3.18	210	45.76 ± 1.73
2	71	52.75 ± 1.88	2.86 ± 0.13	84.56 ± 1.92	153.02 ± 3.21	205	42.62 ± 1.74
3	75	52.80 ± 1.86	2.81 ± 0.13	91.66 ± 1.90	175.37 ± 3.18	215	38.85 ± 1.72
4	74	54.03 ± 1.85	2.82 ± 0.13	86.91 ± 1.88	162.83 ± 3.15	207	41.47 ± 1.71
5	56	51.53 ± 2.14	2.80 ± 0.15	94.76 ± 2.18	174.08 ± 3.64	218	47.37 ± 1.98
6	71	53.61 ± 1.88	2.83 ± 0.13	91.33 ± 1.92	168.48 ± 3.21	218	44.92 ± 1.74
7	74	55.89 ± 1.86	3.02 ± 0.13	76.75 ± 1.90	153.69 ± 3.17	212	37.09 ± 1.72
8	71	51.83 ± 1.88	2.80 ± 0.13	97.84 ± 1.92	172.68 ± 3.21	213	46.83 ± 1.74
9	77	51.79 ± 1.81	2.76 ± 0.13	93.10 ± 1.85	166.74 ± 3.09	209	41.96 ± 1.68
10	79	55.65 ± 1.81	2.81 ± 0.13	85.81 ± 1.84	167.77 ± 3.08	221	38.30 ± 1.67
11	68	52.51 ± 1.93	2.77 ± 0.13	82.70 ± 1.96	152.52 ± 3.28	229	38.80 ± 1.78
12	72	51.10 ± 1.91	2.88 ± 0.13	88.36 ± 1.94	162.96 ± 3.25	209	44.70 ± 1.76
13	75	55.12 ± 1.87	2.89 ± 0.13	90.06 ± 1.91	169.10 ± 3.19	219	43.98 ± 1.73
14	68	51.55 ± 1.91	2.73 ± 0.13	90.43 ± 1.94	166.15 ± 3.25	213	44.93 ± 1.76

Values are estimated marginal means ± model-based standard errors (SEs). HR, heart rate; SE, standard error; n, number of training sessions; bpm, beats per minute. Session peak HR represents the highest heart rate recorded during an individual training session, whereas highest recorded HR represents the maximum value observed across all sessions within the respective horse or exercise modality.

Supplementary Table S2. Descriptive statistics of training sessions by exercise modality.

Workload	*n*	*Time (min)*	*Distance (km)*	*HR average (bpm)*	*Session peak HR (bpm)*	*Highest recorded HR (bpm)*	*HR min. (bpm)*
Walker	58	97.96 ± 2.11	5.57 ± 0.15	71.19 ± 2.15	130.73 ± 3.60	204	43.74 ± 1.95
Lunging	261	22.67 ± 0.98	1.14 ± 0.07	101.45 ± 0.99	166.13 ± 1.66	229	43.66 ± 0.90
lunging + saddle	41	30.94 ± 2.88	1.66 ± 0.20	102.61 ± 2.93	185.59 ± 4.90	210	41.57 ± 2.66
Saddle	424	41.74 ± 0.78	2.31 ± 0.05	92.20 ± 0.79	164.21 ± 1.33	221	40.44 ± 0.72
Test	59	58.81 ± 2.17	2.88 ± 0.15	83.07 ± 2.21	167.92 ± 3.69	217	43.38 ± 2.00
free jumping	160	65.66 ± 1.26	3.41 ± 0.09	81.65 ± 1.29	172.54 ± 2.15	219	43.32 ± 1.17

Values are estimated marginal means ± model-based standard errors (SEs). HR, heart rate; SE, standard error; n, number of training sessions; bpm, beats per minute. Session peak HR represents the highest heart rate recorded during an individual training session, whereas highest recorded HR represents the maximum value observed across all sessions within the respective horse or exercise modality.

Supplementary Table S3. Descriptive statistics of training sessions by monitoring week.

Weeks	*n*	*Time (min)*	*Distance (km)*	*HR average (bpm)*	*Session peak HR (bpm)*	*Highest recorded HR (bpm)*	*HR min. (bpm)*
week 1	52	62.56 ± 2.25	2.88 ± 0.16	81.32 ± 2.29	159.31 ± 3.84	209	34.80 ± 2.08
week 2	69	62.31 ± 1.96	2.92 ± 0.13	79.27 ± 2.00	161.41 ± 3.34	210	37.26 ± 1.81
week 3	67	55.74 ± 1.88	2.89 ± 0.13	87.45 ± 1.91	168.44 ± 3.20	213	37.20 ± 1.73
week 4	65	52.08 ± 2.01	3.05 ± 0.14	91.43 ± 2.05	174.08 ± 3.43	219	38.53 ± 1.86
week 5	64	47.17 ± 2.05	2.43 ± 0.14	91.61 ± 2.09	171.18 ± 3.49	211	42.48 ± 1.89
week 6	66	50.98 ± 2.02	2.42 ± 0.14	87.44 ± 2.06	164.88 ± 3.45	229	40.37 ± 1.87
week 7	51	46.87 ± 2.22	2.31 ± 0.15	88.72 ± 2.26	161.58 ± 3.79	218	41.85 ± 2.06
week 8	40	57.71 ± 2.52	2.79 ± 0.17	91.61 ± 2.57	179.74 ± 4.29	213	39.38 ± 2.33
week 9	55	60.48 ± 2.16	3.16 ± 0.15	88.28 ± 2.20	163.52 ± 3.68	207	44.50 ± 2.00
week 10	48	50.59 ± 2.34	2.70 ± 0.16	90.59 ± 2.38	164.25 ± 3.98	215	41.15 ± 2.16
week 11	60	50.40 ± 2.08	2.58 ± 0.14	92.30 ± 2.12	163.40 ± 3.54	215	49.12 ± 1.92
week 12	59	46.79 ± 2.12	2.16 ± 0.15	95.73 ± 2.16	157.54 ± 3.62	212	52.38 ± 1.96
week 13	64	49.08 ± 2.10	3.37 ± 0.15	94.53 ± 2.15	159.55 ± 3.59	221	50.90 ± 1.95
week 14	62	46.05 ± 2.06	2.91 ± 0.14	91.30 ± 2.10	164.30 ± 5.37	209	40.64 ± 1.91
week 15	47	48.54 ± 2.30	3.25 ± 0.16	90.45 ± 2.35	163.80 ± 3.92	213	44.01 ± 2.13
week 16	60	46.41 ± 2.11	2.87 ± 0.15	94.17 ± 2.15	164.04 ± 3.59	218	49.05 ± 1.95
week 17	49	55.76 ± 2.24	3.38 ± 0.15	90.36 ± 2.28	155.35 ± 3.81	202	45.61 ± 2.07
week 18	25	63.87 ± 3.15	2.82 ± 0.22	69.95 ± 3.21	164.30 ± 5.37	215	39.06 ± 2.92

Values are estimated marginal means ± model-based standard errors (SEs). HR, heart rate; SE, standard error; n, number of training sessions; bpm, beats per minute. Session peak HR represents the highest heart rate recorded during an individual training session, whereas highest recorded HR represents the maximum value observed across all sessions within the respective horse, exercise modality, or monitoring week.

Supplementary Table S4. Estimated energy expenditure per unit of workload (MJ/load) calculated by the Coenen HR and Coenen O_2 methods in 14 three-year-old Czech Warmblood stallions.

Horse	*n*	*Coenen HR*	*Coenen O_2*
1	72	15.45 ± 0.72	13.36 ± 0.65
2	71	12.10 ± 0.73	10.56 ± 0.65
3	75	15.32 ± 0.72	13.28 ± 0.65
4	74	12.55 ± 0.72	11.08 ± 0.64
5	56	15.97 ± 0.83	13.89 ± 0.74

6	71	15.84 ± 0.73	13.81 ± 0.65
7	74	10.31 ± 0.72	8.99 ± 0.65
8	71	15.17 ± 0.73	13.45 ± 0.65
9	77	15.07 ± 0.70	13.17 ± 0.63
10	79	14.46 ± 0.70	12.49 ± 0.63
11	68	11.67 ± 0.75	10.20 ± 0.67
12	72	13.06 ± 0.74	11.45 ± 0.66
13	75	13.98 ± 0.73	12.28 ± 0.65
14	68	13.48 ± 0.74	11.79 ± 0.66

Values are presented as estimated marginal means ± model-based standard errors (SEs). Coenen HR and Coenen O_2 represent two heart-rate-derived methods for estimating energy expenditure. Both methods are based on the same underlying HR recordings. n, number of training sessions; MJ/load, megajoules per complete training session, including warm-up and cool-down.
